# In-Situ Measurement of Fresh Produce Respiration Using a Modular Sensor-Based System

**DOI:** 10.3390/s20123589

**Published:** 2020-06-25

**Authors:** Nandita Keshri, Ingo Truppel, Werner B. Herppich, Martin Geyer, Cornelia Weltzien, Pramod V. Mahajan

**Affiliations:** 1 Department of Horticultural Engineering, Leibniz Institute for Agricultural Engineering and Bioeconomy (ATB), 14469 Potsdam, Germany; nkeshri@atb-potsdam.de (N.K.); itruppel@atb-potsdam.de (I.T.); wherppich@atb-potsdam.de (W.B.H.); mgeyer@atb-potsdam.de (M.G.); cornelia.weltzien@tu-berlin.de (C.W.); 2 Institut für Konstruktion, Mikro- und Medizintechnik, FG Agromechatronik, Technische Universität Berlin, Straße des 17. Juni 135, 10623 Berlin, Germany

**Keywords:** Dynamic Controlled Atmosphere (DCA) storage, Respiratory Quotient (RQ), DCA-RQ, apples, strawberries, O_2_ and CO_2_ sensors

## Abstract

In situ, continuous and real-time monitoring of respiration (R) and respiratory quotient (RQ) are crucial for identifying the optimal conditions for the long-term storage of fresh produce. This study reports the application of a gas sensor (RMS88) and a modular respirometer for in situ real-time monitoring of gas concentrations and respiration rates of strawberries during storage in a lab-scale controlled atmosphere chamber (190 L) and of Pinova apples in a commercial storage facility (170 t). The RMS88 consisted of wireless O_2_ (0% to 25%) and CO_2_ sensors (0% to 0.5% and 0% to 5%). The modular respirometer (3.3 L for strawberries and 7.4 L for apples) consisted of a leak-proof arrangement with a water-containing base plate and a glass jar on top. Gas concentrations were continuously recorded by the RMS88 at regular intervals of 1 min for strawberries and 5 min for apples and, in real-time, transferred to a terminal program to calculate respiration rates ($R_{O_{2}}$ and $R_{{\mathrm{CO}}_{2}}$) and RQ. Respiration measurement was done in cycles of flushing and measurement period. A respiration measurement cycle with a measurement period of 2 h up to 3 h was shown to be useful for strawberries under air at 10 °C. The start of anaerobic respiration of strawberries due to low O_2_ concentration (1%) could be recorded in real-time. $R_{O_{2}}$ and $R_{{\mathrm{CO}}_{2}}$ of Pinova apples were recorded every 5 min during storage and mean values of 1.6 and 2.7 mL kg^−1^ h^−1^, respectively, were obtained when controlled atmosphere (CA) conditions (2% O_2_, 1.3% CO_2_ and 2 °C) were established. The modular respirometer was found to be useful for in situ real-time monitoring of respiration rate during storage of fresh produce and offers great potential to be incorporated into RQ-based dynamic CA storage system.

## 1. Introduction

Respiration (R) of fresh fruit and vegetables is an indicator of ongoing metabolic processes after harvest. The respiratory quotient (RQ) is defined as the ratio of the CO_2_ produced to the O_2_ consumed by respiration. R and RQ of fresh produce have pronounced influences on postharvest quality and shelf-life and are, therefore, key parameters to be controlled for the extension of shelf life. Quantifying respiration in terms of rates of O_2_ consumption and CO_2_ production ($R_{O_{2}}$ and $R_{{\mathrm{CO}}_{2}}$) is an important and helpful tool for optimising the design of packaging systems and maintaining optimum conditions inside storage systems. To quantify respiration of stored fresh produce, the respective respiration-induced changes in the headspace concentrations of O_2_ and CO_2_ is measured over time. This is normally done by gas chromatography [1,2,3] or preferably by handheld or table-top gas analyzers due to their compact size, ease of handling and lower cost compared to gas chromatographs [4]. However, such analyzers have the drawback of taking up a certain amount of gas sample (3 mL to 15 mL) every time when measuring, thereby creating low pressure inside the storage space. Another disadvantage of such analyzers is that they produce discrete data and, thus, discontinuous analyses [4]. Most of the methods reported so far require fruit samples to be removed from the actual in situ storage environment for respiration measurement. This manual and time-consuming sampling needs equilibration of produce to the storage atmosphere [5], which may affect the physiological responses of fresh produce. Moreover, the measurement intervals should be representative of the changing respiration pattern and should be chosen with due care [6]. Therefore, there is a need to measure respiration activity directly inside the storage environment, to allow continuous in situ monitoring of respiration in real-time. 

Recent reports recommended placing sensors directly inside the storage or the packaging system to measure O_2_ and/or CO_2_ concentrations, thus enabling dynamic real-time control of gas compositions [3,7,8,9,10,11]. Ortiz [12] applied a system developed for analysis of human respiration to continuously record the dynamics of in situ respiration of fresh produce and rated it as a powerful but cost-intensive technique. An on-line system was proposed by Mahajan [3] with O_2_ and CO_2_ sensors placed directly on the metal lid of a glass jar. This system, however, was prone to gas leakage due to the various attachments on the lid, especially when working at low O_2_ concentrations. Løkke [10] emphasized the continuous measurement of respiration rate and applied wireless sensor networks for continuous measurement of respiration rate in small jars of 1 L. Such a system, however, was unable to determine O_2_ concentration lower than 5%.

Controlled atmosphere (CA) storage has become the most adopted technology worldwide to extend shelf life of fresh produce. More recently, use of a dynamic, rather than a static, controlled atmosphere has gained attention. In dynamic controlled atmosphere (DCA) systems, the storage conditions are registered by monitoring the physiological response of fruits such as in a dynamic controlled atmosphere-respiratory quotient (DCA-RQ) system [13,14,15,16,17]. A limitation of a DCA-RQ system is leakage of the storage facility because a small leak from the storage room will lead to erroneous values of RQ [18]. Bessemans [13] introduced a new automatic DCA control system based on online real-time RQ measurements that took into account leakage in a predictive model. However, since the variability of the gas tightness of a commercial CA storage is not predictable, the mathematical model could be prone to errors. In a DCA-RQ system, the entire storage is isolated from a gas control system so the concentration of O_2_ and CO_2_ can be measured and RQ calculated. The estimated RQ is then used to control the O_2_ concentration [11,16,17,19,20]. The limitation of such a system is that the entire storage room must be temporarily isolated for several hours (from 4 h to 24 h) so no O_2_ and CO_2_ gas control takes place during this period. This may lead to stress induction on stored fruits due to changing atmospheric conditions. Moreover, RQ measurement is performed at certain time intervals so that further delay in processing the data and implementing the correct measures may affect fruit physiology permanently. An advancement to the current system was demonstrated by Brackmann [14] in which a chamber, instead of the whole storage cell, was isolated for RQ measurement for 24–36 h. However, such a system requires a capital investment and seems expensive to operate. Moreover, the isolated chamber itself might be prone to leakage leading to erroneous values of RQ. 

Schaefer [16] demonstrated a control system that included an enclosure that could be placed within a CA room to store a representative sample of the samples and isolate the enclosure for measuring RQ, externalising control accordingly. However, such a system did not use a wireless sensor system for continuous and real-time monitoring. Recently, wireless sensor networks have often been used because they provide continuous information on important factors such as CO_2_ and O_2_ concentrations, temperature, humidity and atmospheric pressure, all of which influence respiration. The objective of the present study was to assess the performance of a mobile modular O_2_ and CO_2_ gas sensor system based on wireless sensors, and a leak-proof respirometer for continuous and real-time in situ monitoring of respiration and RQ in both a lab-scale and a commercial CA storage facility for strawberries and apples, respectively. The aim of the study was to showcase the novel technology of a mobile sensor-based system to wirelessly, continuously and in real-time measure respiration rate and RQ of stored produce in situ.

## 2. Materials and Methods

In the initial course of testing the in-situ measurement setup, fresh strawberries were selected, while subsequent testing was performed with Pinova apples in a commercial CA storage room.

### 2.1. Plant Materials and Controlled Atmosphere (CA) Facility

**Figure 1:**
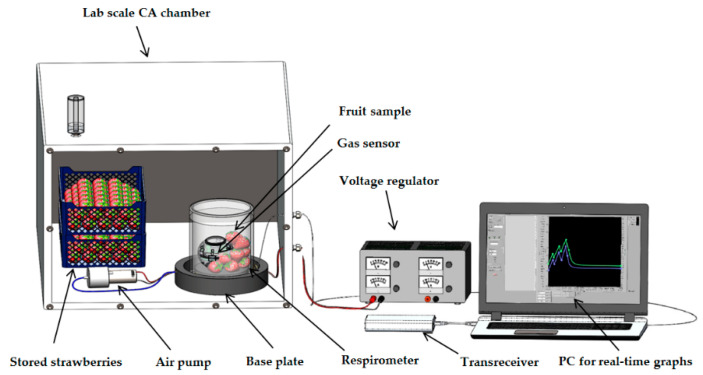
Scheme of the lab-scale in situ respiration and respiratory quotient (RQ) measurement setup, consisting of the controlled atmosphere (CA) chamber and the measurement equipment.

**Figure 2:**
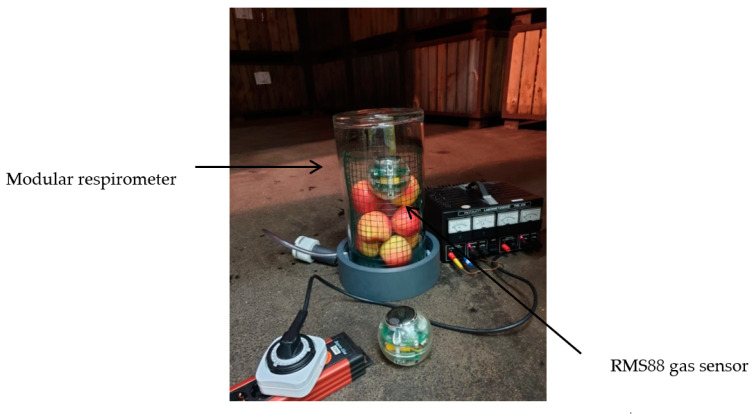
The modular respirometer filled with Pinova apples along with the RMS88 gas sensor installed inside a commercial CA storage unit at 2% O_2_, 1.3% CO_2_ and 2 °C.

Fresh strawberries, from Brandenburg, Germany harvested in the month of May were selected as sample produce for in situ measurements due to their high respiration activity, which made it easy to realise fast changes in R and RQ in a short time. Strawberries (1.2 kg and 1.5 kg in Experiment I and II, respectively) were stored in a temperature-controlled 190 L-lab scale CA chamber (Figure 1; Frigotech GmbH, Landsberg, Germany) at varying atmospheric conditions (Table 1, Experiment I and II). A further testing of in situ measurements (Table 1, Experiment III) was performed in a commercial CA storage room (6.6 m × 15.2 m × 7.0 m; Havelfrucht GmbH, Werder, Germany) with Pinova apples harvested from orchards in the Werder area in November 2019 (Figure 2). Experiments with Pinova apples were performed at CA room conditions set to 2% O_2_, 1.3% CO_2_ and 2 °C temperature for 32 d of storage (from 05.11.2019 until 06.12.2019). 

### 2.2. Construction of Respiration Measuring Gas Sensor

**Figure 3:**
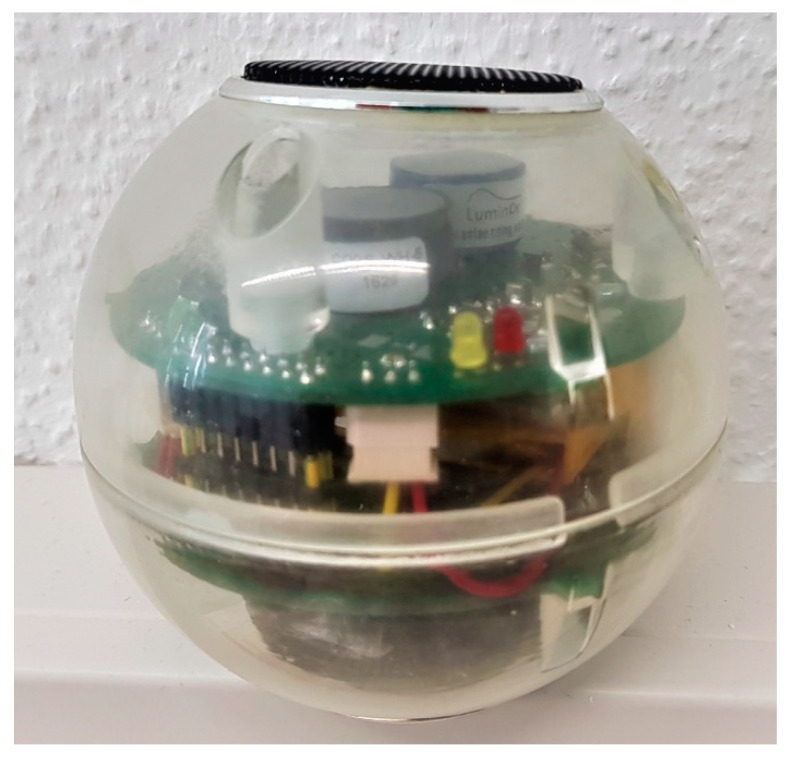
Schematic of the respiration measuring sphere (RMS88).

The respiration measuring sphere or RMS88 gas sensor (Figure 3) as described by Keshri [6] consisted of two circular printed circuit boards (diameter: 76 mm, mass: 191 g) and two Li-polymer rechargeable batteries mounted in a hollow transparent resin sphere (diameter: 88 mm; Synthene, Pont-Sainte-Maxence, France). At two opposite positions of the sphere, a metal grating was glued into openings to allow for free gas diffusion. The printed circuit board of the RMS88 housed sensors for O_2_ and CO_2_ measurements (SST Sensing Ltd., Coatbridge, UK). A fluorescence based optical O_2_ sensor allowed a measurement range of 0–25% (resolution: 0.01% and accuracy: 2% of full scale), while nondispersive infrared CO_2_ sensors measured in the range of 0–5000 ppm (resolution: 1 ppm and accuracy: ±50 ppm ±3% of reading) and 0–50,000 ppm (resolution: 10 ppm and accuracy: ±70 ppm ±5% of reading). The printed circuit board also housed an ATMEGA328P microcontroller, a real-time clock, 256 kByte F-RAM, an 869 MHz transceiver for wireless data transfer, a charging circuit for the battery and a reset button. A mini USB port provided communication with a PC and allowed charging of the battery. Additionally, both CO_2_ sensors measured relative humidity (RH) and temperature. 

As soon as the RMS88 was activated, it started to measure, store and wirelessly transfer O_2_, CO_2_, temperature and RH data. The RMS88 could measure at intervals of 1, 2, 3, 4, 5, 6, 10, 12, 15, 20, 30 or 60 min. Applying a measuring interval of 5 min, the RMS88 could store data sets for up to 100 d if fully charged. Raw data (measured gas concentrations, temperature and RH), as well as processed data (respiration rates and RQ), could be obtained wirelessly on an in-house developed LabView-based (National Instruments Corporation, Austin, TX, USA) software “Gassensor” for real-time calculation of respiration rates (Section 2.4). To stop the measurements, the RMS88 was again connected to the terminal program. Data stored in memory could be retrieved later after stopping the RMS88. However, data transferred to the PC in real-time on the software could be downloaded and saved in Excel format for future analysis (if required).

### 2.3. Modular Respirometer

Each of two modular respirometers of different sizes (3.3 L for strawberries; 7.4 L for apples) with leak-proof design as described by Keshri [6], consisted of a base plate with a raised platform in the centre surrounded by a channel for water filling for leak-proofing and for covering the base plate with a removable cylindrical glass cover. The raised platform accommodated the RMS88 gas sensor and the fruit sample. The base plate also had inlet and an outlet tubes for gas flushing. In this study, respiration measurements were done in cycles. Each cycle consisted of a fixed flushing period of 1 h and a varying measurement period (Table 1). Gas flushing was performed in each cycle using a timer-switched air pump (flow rate 5 L min^−1^). During the 1 h flushing period, R and RQ measurements were stopped and the air pump was switched on. This was required to purge gas from the respirometer and bring in new gas from the CA storage for the next cycle. During the measurement period, the air pump was switched off, thus isolating the respirometer from the CA storage. The changes in O_2_ and CO_2_ concentrations during the measurement period were used to calculate R and RQ. After each measurement period, the air pump was automatically switched on using a timer clock to start the flushing period. During the whole experimental period, the RMS88 was recording and sending O_2_ and CO_2_ concentration data in real-time to the PC. However, only the data recorded during the measurement period were used for the calculation of R and RQ (Section 2.4).

### 2.4. Calculation of Respiration Rate

Data collected by the RMS88 at intervals of 1 min (Experiment I to II) and 5 min (Experiment III) were stored in the memory of the RMS88 and also directly transmitted to a PC to visualize the parameters in real-time and to calculate respiration rates using the Gassensor software. Respiration rates ($R_{O_{2}}$ and $R_{{\mathrm{CO}}_{2}}$ in mL kg^−1^ h^−1^) and RQ were calculated as

(1) $$R_{O_{2}}=\frac{{\Delta O}_{2}}{100*\Delta t}\times \frac{V_{\mathrm{net}}}{m_{p}}$$

(2) $$R_{{\mathrm{CO}}_{2}}=\frac{{\Delta \mathrm{CO}}_{2}}{100*\Delta t}\times \frac{V_{\mathrm{net}}}{m_{p}}$$

(3) $$\mathrm{RQ}=\frac{R_{CO_{2}}}{R_{O_{2}}}$$

where ΔO_2_ and ΔCO_2_ denote the changes of the respective gas concentration in the chamber (%), Δt is the time interval (h), $V_{\mathrm{net}}$ the free gas volume inside the respirometer (mL) and $m_{p}$ the mass of the product (kg). RQ was calculated as the ratio of $R_{{\mathrm{CO}}_{2}}$ and $R_{O_{2}}$. From the preliminary trials, it was observed that the atmosphere inside the respirometer needed some time to equilibrate after each flushing; therefore an initial 30 min period was set as a wait time before beginning the respiration rate calculations.

## 3. Results

### 3.1. Respiration Measured over Varying Respiration Measurement Periods (Experiment I)

**Figure 4:**
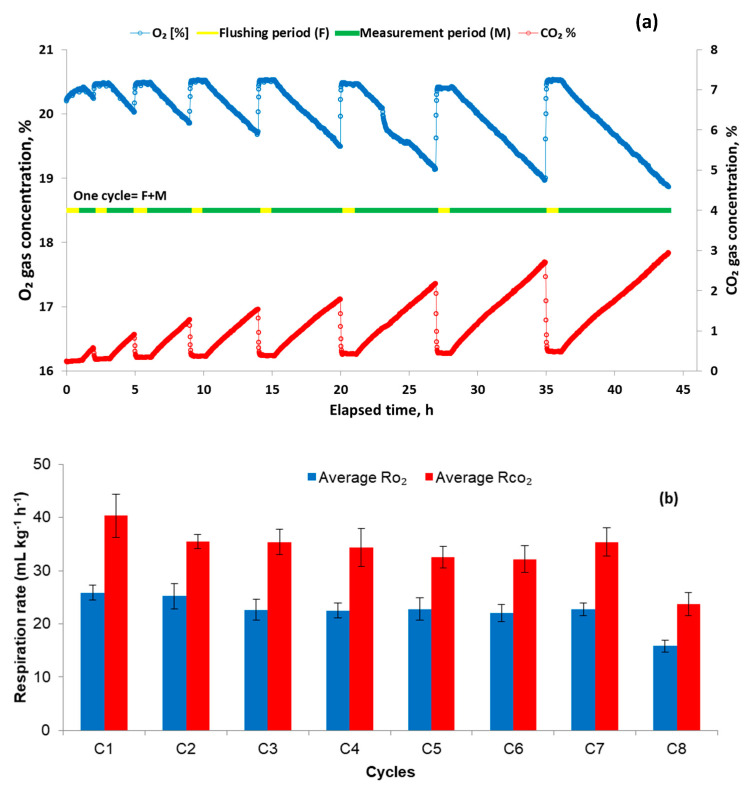
(**a**) Variations in CO_2_ and the O_2_ concentrations within the respirometer filled with strawberries due to respiration and air-flushing. (**b**) Calculated mean real-time respiration rates ($R_{O_{2}}$ and $R_{{\mathrm{CO}}_{2}}$) during the measuring period in subsequent cycles under air at 10 °C.

Figure 4a shows changes in gas composition inside the respirometer during storage. Flushing of the respirometer using the air pump for 1 h was shown to be sufficient to bring in fresh air from the CA storage chamber. Once flushing had stopped, the decrease in O_2_ and increase in CO_2_ concentrations due to respiration of the stored strawberry sample inside respirometer was evident. Figure 4b shows the calculated mean $R_{O_{2}}$ and $R_{{\mathrm{CO}}_{2}}$ during the various measurement periods, which were step-wisely prolonged by 1 h in subsequent cycles. Varying respiration measurement periods were used to optimise cycle time for respiration measurement of strawberries with the modular sensor-based system. A short measuring period of 1 h (C1 in Figure 4b) resulted in high fluctuations in $R_{{\mathrm{CO}}_{2}}$ values calculated every minute (SD of ± 4.1 mL kg^−1^ h^−1^ over the cycle; data not shown). Prolonging the measuring period from 2 h to 7 h (C2 to C7 in Figure 4b) had only a slight effect on mean respiration rates. Mean $R_{O_{2}}$ of 25.2 mL kg^−1^ h^−1^ and 22.7 mL kg^−1^ h^−1^ and $R_{{\mathrm{CO}}_{2}}$ of 35.4 mL kg^−1^ h^−1^ were observed at 2 h and 7 h measuring periods, respectively. Long-term measuring periods (8 h), however, resulted in lower mean $R_{O_{2}}$ (15.8 mL kg^−1^ h^−1^) and $R_{{\mathrm{CO}}_{2}}$ (23.7 mL kg^−1^ h^−1^) values. This resulted from the high CO_2_ concentrations, which accumulated inside the respirometer due to active fruit respiration and, in turn, depressed the respiration rate of the strawberries. Accumulation of high CO_2_ concentrations around fresh produce often induces off flavors and tissue breakdown [21]. Therefore, the respiration measurement period should be chosen with care to avoid accumulation of high CO_2_ concentrations [22,23]. 

An increase of CO_2_ concentrations from atmospheric levels to 0.5–1% has been reported as sufficient to calculate the respiration rates of fresh produce [2]. In general, $R_{{\mathrm{CO}}_{2}}$ values of strawberries were obtained in the range of 25 mL kg^−1^ h^−1^ to 50 mL kg^−1^ h^−1^ for aerobic respiration at 10 °C [24,25]. It is proposed that a measuring period of 2 to 3 h for strawberries was enough to determine respiration and RQ accurately in the present experiment. A longer measuring period may cause a large change in the O_2_ and CO_2_ partial pressure, which, in turn, may affect the physiology of the fruit. In this study, the use of a modular sensor-based system allowed variations in respiration measurement periods to optimise the period in real-time. Therefore, flushing of the respirometer with air from the CA chamber at defined intervals created a new opportunity for in situ monitoring of actual respiration activity of fresh produce. With the use of such a system, variations in respiration measurement period can be made in real time based on actual storage conditions.

In the current study, the modular system was always on, even when R and RQ were not being measured. The cycle of purging the gas and measuring respiration during isolation mode was continuous and the operation of the system could not be stopped. However, it is possible to prolong the cycle, which may need different time intervals for purging and R and RQ measurements. However, in this case, the respirometer must be optimized in terms of the amount of fruit and respirometer volume so that no excessive CO_2_ accumulates.

### 3.2. Respiration Measured under Controlled Atmosphere (Experiment II)

**Figure 5:**
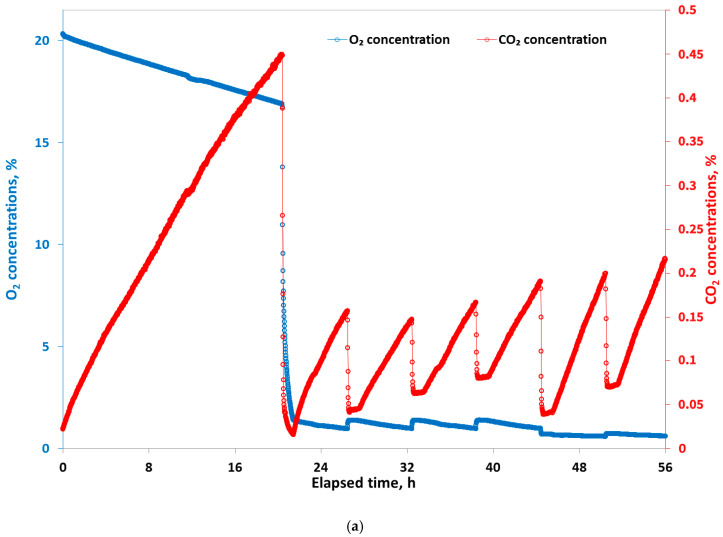

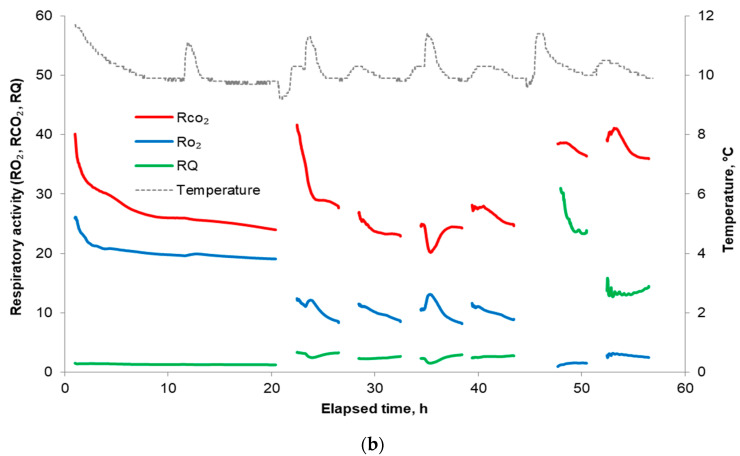
(**a**) Change in gas composition over time during a 56 h storage period of fresh strawberries; (**b**) in situ real-time respiration rate and RQ calculated for strawberries under air and at low O_2_ (1%) at 10 °C inside respirometer.

Similar to the results of previous experiments on fresh strawberries under air, mean $R_{O_{2}}$ and $R_{{\mathrm{CO}}_{2}}$ were 20.2 ± 1.2 mL kg^−1^ h^−1^ and 27 ± 2.8 mL kg^−1^ h^−1^ (Figure 5b), respectively, and the RQ (Figure 5b) was 1.33 after 20 h of storage in a CA chamber for 1.5 kg of strawberries. Lowering the O_2_ concentrations from 21% to 2% (Figure 5b) diminished $R_{O_{2}}$ to 10.5 ± 1.3 mL kg^−1^ h^−1^ but temporarily increased $R_{{\mathrm{CO}}_{2}}$ to 31.2 ± 3.4 mL kg^−1^ h^−1^ probably due to the accumulation of high CO_2_ concentrations (4.5%) in the 1st measurement cycle under air (18 h measurement period). The accumulated CO_2_ may not have been completely flushed out from the spherical RMS88 gas sensor and was released back to the respirometer, falsely indicating as CO_2_ production due to respiration of the strawberries. Lowering the O_2_ concentration to 1% after 45 h resulted in a sudden rise in RQ, sensitively indicating the switch from aerobic to anaerobic respiration. Woodward [26] also reported O_2_ concentrations of 1%, and lower, as critical for fruit and vegetables, including strawberries. Such a sudden rise and starting point of the anaerobic respiration point of stored produce is important. 

In situ measurements using the modular sensor-based system were shown to be useful for real-time detection of physiological changes in stored produce. The air temperature inside the respirometer fluctuated during measurements (Figure 5b). The sensor electronics produced a small amount of heat (about 10 mW), which led to a small increase of the measured temperature of approx. ≤2 °C, especially noticeable when there was no air flow inside the respirometer. However, such small changes in temperature in the sphere (~2 °C; Figure 5b) did not influence the respiration rate of stored produce.

### 3.3. Respiration Measured in Commercial Apple CA Storage (Experiment III)

**Figure 6:**
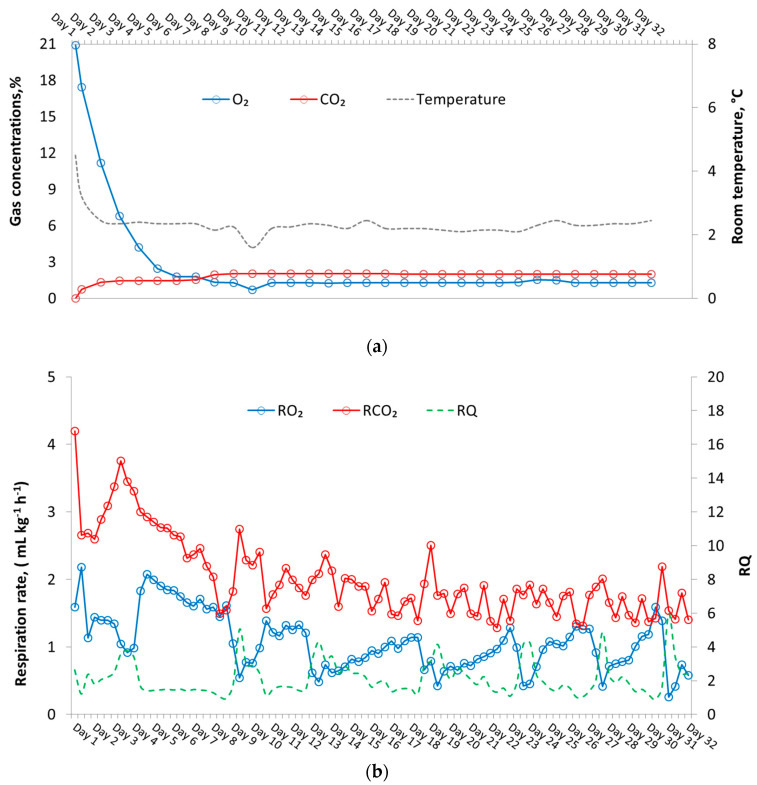
(**a**) Controlled atmosphere conditions monitored inside a CA storage unit over a storage period of 32 days with Pinova apples; (**b**) mean in situ respiration rate calculated using a modular respirometer and RMS88 for Pinova apples stored at CA storage conditions of 2% O_2_, 1.3% CO_2_ at 2 °C.

Approximately 8 d after the closure of the CA room with the Pinova apples, atmospheric conditions had stabilised at 2 °C, close to the desired setting (2% O_2_, 1.3% CO_2_). CA conditions then remained constant until the CA room was re-opened after a total of 32 d (Figure 6a). The respirometer worked at 8 h-cycles with continuous and real-time R and RQ measurements every 5 min. Thus, three sets of mean respiration results could be obtained each day (Figure 6b). A longer recording interval (5 min) and respiration measurement period (8 h) were used because of the slow respiration of apples. During the initial 8 d of storage, mean maximum $R_{O_{2}}$ and $R_{{\mathrm{CO}}_{2}}$ were 2.2 ± 0.3 and 4.2 ± 0.6 mL kg^−1^ h^−1^, respectively (Figure 6b). Mean $R_{O_{2}}$ and $R_{{\mathrm{CO}}_{2}}$ stabilised at 1.6 and 2.7 mL kg^−1^ h^−1^, respectively, when constant CA conditions were established. These rates closely reflect those measured by Mitcham [27] under similar conditions. RQ observed during measurement showed peaks reaching a value of 4.0. However, such a short time increase in RQ is not harmful to apples and, in fact, results in better maintenance of postharvest quality [14]. The modular sensor-based system used in this study was able to detect such variations in RQ during storage in real-time. The O_2_ concentration was controlled well above the critical limit for apples (0.2–0.4%) [28]. Further efforts are needed to use this experimental setup for a DCA system and monitor respiration rate and RQ over a longer period and to compare the results with chlorophyll fluorescence-, or ethanol-based DCA storage system, in a commercial setup.

The respirometer is not limited to its current size, which can be increased if required. In addition, multiple units of the respirometer and the RMS88 system can be installed at multiple locations of a commercial storage chamber in order to monitor samples at different locations.

## 4. Conclusions

This study demonstrated a modular system for continuous in situ and real-time monitoring of both O_2_ and CO_2_-based respiration rates and RQ in a lab-scale and a commercial-scale CA storage facility. For produce such as strawberries and apples, successful application of this system enabled the continuous monitoring of concentrations of relevant gases and, more importantly, of respiration activity and RQ under a wide range of different storage conditions and over the entire storage period. The leak-proof and mobile design of the modular sensor-based system allowed wireless monitoring of the start of anaerobic activity in real-time. Altogether, these features recommend the modular respirometer for incorporation in an advanced DCA monitoring and control system. It also has great potential for adoption and integration into existing CA control systems to get additional important information on the quality of valuable stored fresh produce. Both are of great economic and ecological relevance for producers and consumers.

## Figures and Tables

**Table 1:** Details of in situ measurements of respiration and RQ under lab-scale controlled atmosphere (CA) storage (Experiment I and II) and in a commercial CA room (Experiment III). Flushing period was always 1 h.

Experiment	Produce	Sample Mass (g)	Temperature (°C)	Gas Composition	Flushing Cycles		Storage Duration
					Measurement Period (h)	Number of Cycles/Day	
I	Strawberries	280	10	Air	Varying	Varying	44 h
II	Strawberries	260	10	Air, then 1% O_2_	5	4	56 h
III	Pinova apples	1436	2	2% O_2_ + 1.3% CO_2_, N_2_-balanced	7	3	32 d
